# A local difference in blood–brain barrier permeability in the *caudate putamen* and *thalamus* of a rat brain induced by focused ultrasound

**DOI:** 10.1038/s41598-020-76259-z

**Published:** 2020-11-06

**Authors:** Hyungkyu Huh, Tae Young Park, Hyeon Seo, Mun Han, Byeongjin Jung, Hyo Jin Choi, Eun-Hee Lee, Ki Joo Pahk, Hyungmin Kim, Juyoung Park

**Affiliations:** 1 Medical Device Development Center, Daegu-Gyeongbuk Medical Innovation Foundation, Daegu, Korea; 2 Center for Bionics, Korea Institute of Science and Technology, Seoul, Korea; 3 Division of Bio-Medical Science & Technology, KIST School, Korea University of Science and Technology, Seoul, Korea

**Keywords:** Blood-brain barrier, Biomedical engineering

## Abstract

A blood–brain barrier (BBB) opening induced by focused ultrasound (FUS) has been widely studied as an effective way of treating brain diseases. We investigate the effect of ultrasound’s incidence angle at *caudate putamen* (*Cp*) and *thalamus* (*Th*) of the rat brain by inducing the same power of focused ultrasound that corresponds to the acoustic pressure of 0.65 MPa in free field. The BBB permeability (K_trans_) was quantitatively evaluated with dynamic contrast-enhanced magnetic resonance imaging. The group averaged (n = 11) maximum K_trans_ at *Cp* (0.021 ± 0.012 min^−1^) was 1.39 times smaller than the K_trans_ of *Th* (0.029 ± 0.01 min^−1^) with p = 0.00343. The group averaged (n = 6) ultrasound’s incidence angles measured using the computed tomography image of rat skulls were compared with the maximum K_trans_ and showed a negatively linear relation R^2^ = 0.7972). The maximum acoustic pressure computed from the acoustic simulation showed higher average acoustic pressures at *Th* (0.37 ± 0.02 MPa) compared to pressures at *Cp* (0.32 ± 0.01 MPa) with p = 0.138 × 10^−11^. More red blood cell were observed at the *Th* region compared to the *Cp* region in the tissue staining. These results indicate that localized characteristics of the sonication target within the subject should be considered for safer and more efficient BBB disruption induced by FUS.

## Introduction

Despite the increasing number of potent neurologically active substances and drugs, the treatment of central nervous system (CNS) disorders is still very challenging as the delivery of therapeutic agents is limited by the blood–brain barrier (BBB)^1–6^. BBB, unique properties of the microvasculature of the CNS, is known to prevent the transportation of molecules from the circulating blood into the brain parenchyma. As a result, more than 98% of the therapeutic agent normally cannot penetrate BBB, limiting the treatment of brain disorders, such as Alzheimer’s disease, Parkinson’s disease, strokes, and tumours^7,8^.

A non-invasive, localized BBB disruption (BBBD) using a short burst of focused ultrasound (FUS) incorporated with the intravenously injected microbubbles has been previously reported to overcome these limitations^9–11^. In addition, pre-clinical studies reported the successful delivery of several chemotherapeutic drugs, such as doxorubicin (DOX), cytarabine, and 1,3-bis (2-chloroethyl)-1-nitrosourea (BCNU) in rodent brain disease models^12–14^. Based on these promising pre-clinical results, clinical trials are ongoing to verify the efficacy of the FUS-induced BBBD for brain tumours (NCT03551249, NCT03714243, NCT03616860), Alzheimer’s disease (NCT03119961, NCT03671889, NCT03739905), and Parkinson’s disease (NCT03608553).

The effect of sonication parameters, including ultrasound frequency, acoustic pressure, amplitude, pulse length, sonication duration, and the dose and size of the microbubbles on the degree of BBBD has been widely studied, and their correlations are well established for better control of BBBD^14–18^. A previous pre-clinical study by Treat et al.^14^ reported a location dependence of the FUS threshold for the consist BBBD at anterior quadrants and the posterior quadrants of rat brain. Similar trends were also reported in a pre-clinical study^13^, where higher acoustic power was used at the anterior quadrant regions compared to the posterior quadrant regions to achieve a consistent degree of BBBD in the rat model. Additionally, previous ex-vivo studies have reported a linear dependency of the ultrasound transmission on animal body mass^6,19^, as the thickness of the skull increases with the body mass of the healthy subject. These results emphasize the importance of individual physiological characteristics of FUS-induced BBBD. However, the effect of localized differences on BBB permeability after FUS treatment has not been quantitatively reported.

This study investigates the correlation between the degree of BBBD using dynamic contrast-enhanced MRI (DCE-MRI) and ultrasound’s incidence angle by inducing the same level of FUS on two different regions of a rat brain (*caudate putamen; Cp, thalamus; Th*). In addition, acoustic simulations based on computed tomography (CT) images and haematoxylin and eosin (H&E) tissue staining were performed to analyse the difference of acoustic pressure and tissue damages on the two target regions, respectively.

## Result

### BBB permeability

**Figure 1:**
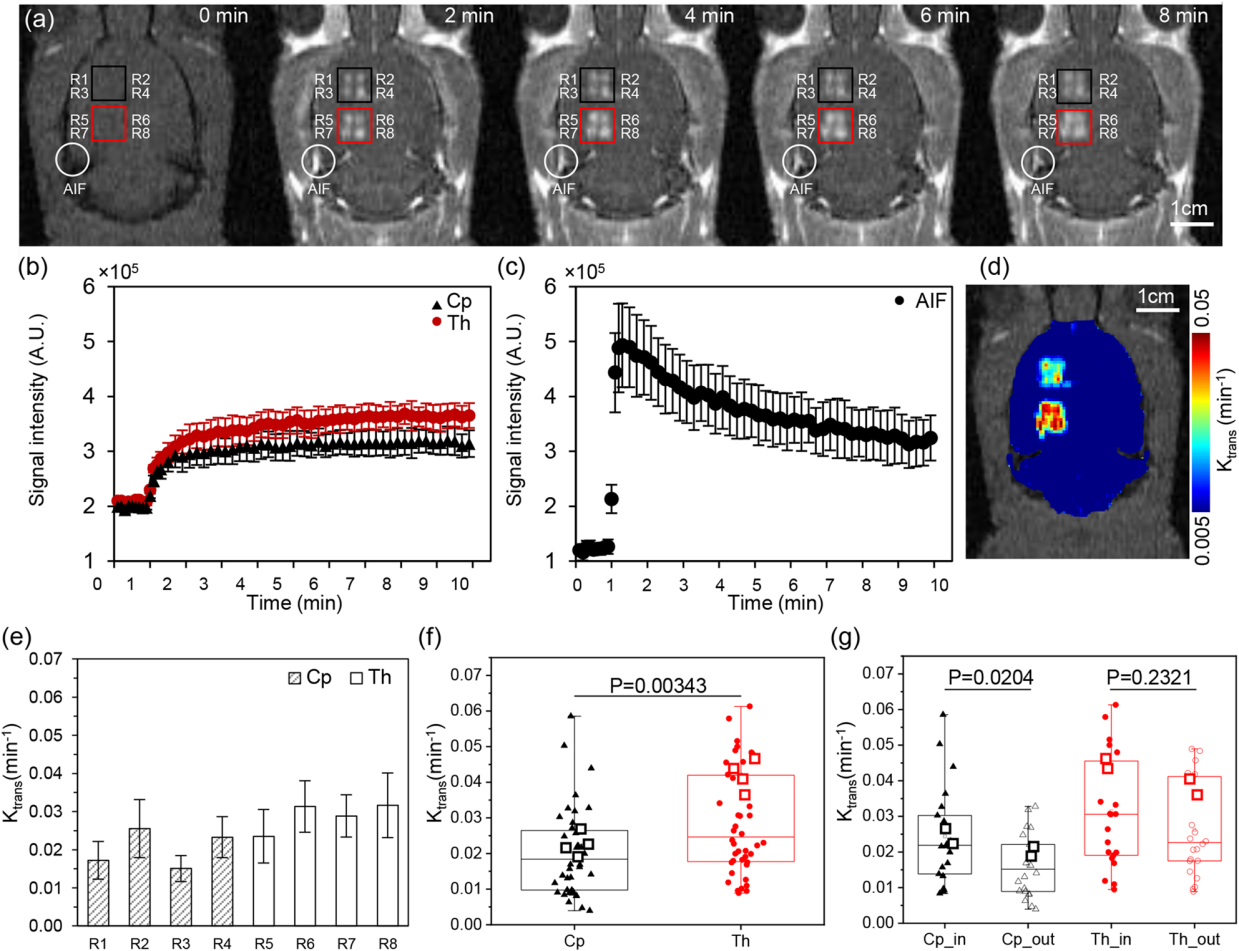
(**a**) A representative DCE image of a rat brain before and 2, 4, 6 and 8 min after sonication. Black and red box indicate *Cp* and *Th*, respectively. An arterial input function was measured at an artery near the sonication site marked as a white circle. A scale bar indicates 1 cm. An average signal intensity of (**b**) *Cp* (triangle), *Th* (circle), and (**c**) arterial input function according to time. (**d**) A K_trans_ map after FUS induced sonication. (**e**) Group averaged (n = 11) maximum K_trans_ of each ROI, where grey and blank bar indicates ROI at *Cp* and *Th*, respectively. (**f**) A averaged maximum K_trans_ of *Cp* (triangle) and *Th* (circle). (**g**) A box plot grouped *Cp*_in (R2, R4) *Cp*_out (R1, R3), *Th*_in (R6, R8) and *Th*_out (R5, R7) marked with a triangle, empty triangle, circle, and empty circle, respectively. A representative DCE case is marked with a square.

BBB permeability (K_trans_) of each region of interest (ROI) after BBB disruption at the *Cp* (R1 ~ R4) and *Th* (R5 ~ R8) regions were computed using DCE-MRI. Figure 1a shows representative DCE-MR images at 0, 2, 4, 6, and 8 min. An MR contrast agent (CA) Gd-DTPA (Magnevist, Bayer HealthCare Pharmaceuticals, Germany), was injected at 1 min. The red and black box indicates each of four ROIs in the *Cp* and *Th* regions of the brain. The corresponding averaged signal intensity within each box, according to the time, is depicted in Fig. 1b. The signal intensity rapidly increases during the first half-minute after contrast agent injection and saturates around six minutes. The spatially averaged signal intensity after the saturation was increased 1.5 and 1.7 times at *Cp* and *Th* after CA injection, respectively. The temporal variation of the signal intensity at the artery (marked circle in Fig. 1a) was measured to derive the individual arterial input function (Fig. 1c). The increased signal intensity rapidly decreased after 30 s at the artery owing to CA washout. Figure 1d shows the corresponding K_trans_ map. The averaged K_trans_ was 0.030 ± 0.025 min^−1^ at the *Th* and 0.059 ± 0.039 min^−1^ at the *Cp*.

The group averaged maximum K_trans_ (n = 11) of each ROI is shown in Fig. 1e. Values at R1–R8 were 0.017 ± 0.005, 0.026 ± 0.008, 0.015 ± 0.003, 0.023 ± 0.005, 0.023 ± 0.007, 0.031 ± 0.007, 0.029 ± 0.006, and 0.032 ± 0.009 min^−1^, respectively. The highest and lowest groups averaged maximum K_trans_ was found at R8 and R3, respectively, and the group averaged maximum K_trans_ of *Cp* (0.021 ± 0.012 min^−1^) was 1.39 times smaller than the K_trans_ of *Th* (0.029 ± 0.014 min^−1^), with p = 0.00343 (Fig. 1f). In general, medial ROI (even number) showed higher maximum K_trans_ compared to the corresponding lateral ones (odd number). The group averaged maximum K_trans_ of medial ROI (0.024 ± 0.013 min^−1^) at *Cp* were 1.51 times higher than the lateral ROI at *Cp* (0.016 ± 0.009 min^−1^) with p = 0.0204. Meanwhile, the group averaged maximum K_trans_ of medial ROI (0.027 ± 0.013 min^−1^) at *Th* were 1.41 times higher than the lateral ROI at *Th* (0.019 ± 0.012 min^−1^), with p = 0.232 as shown in Fig. 1g.

### The incidence angle of the ultrasound beam

**Figure 2:**
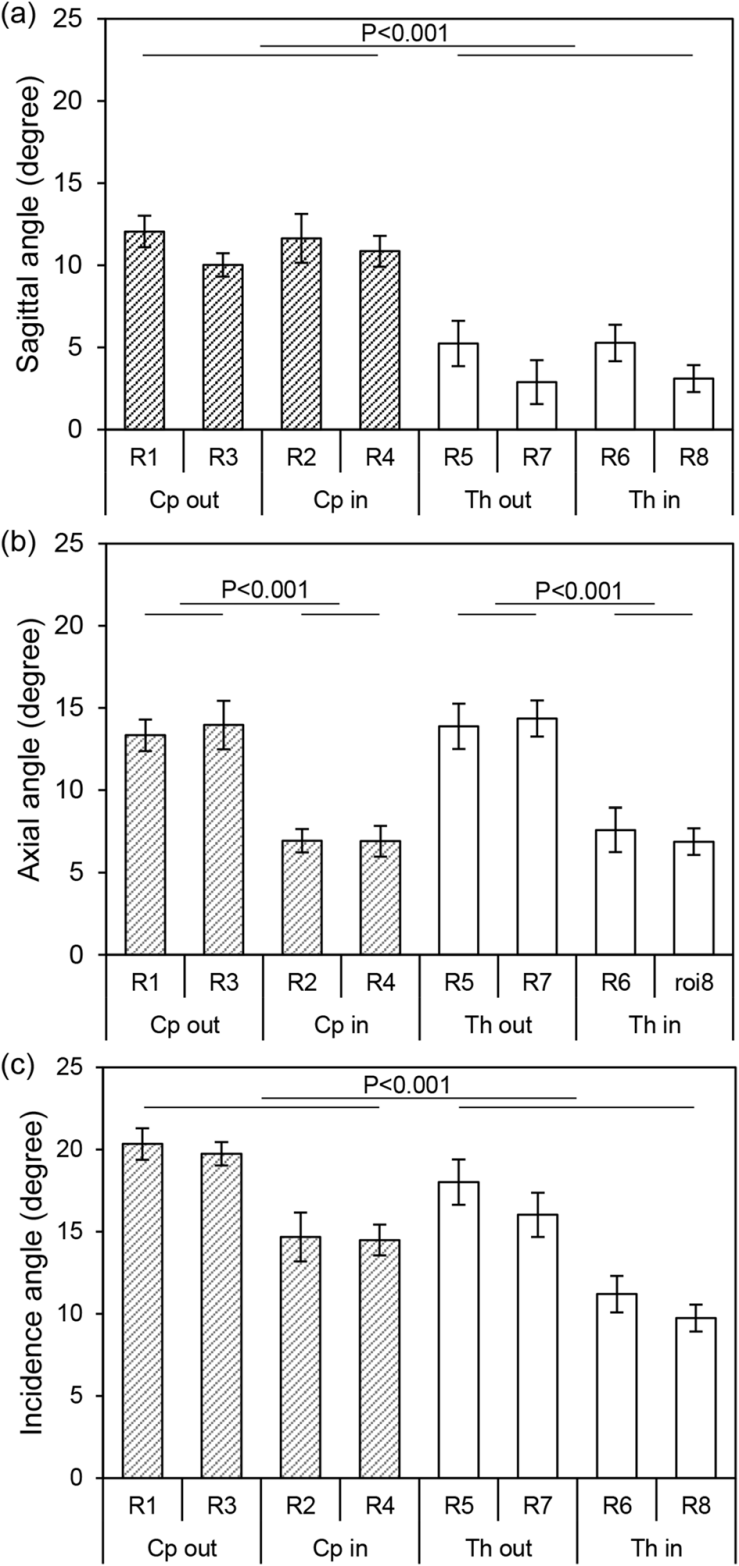
A group averaged angle (n = 6) of each ROI measured at (**a**) sagittal view, (**b**) axial view, and (**c**) incidence angle from 3D reconstructed surface. Grey and blank bar indicate *Cp* and *Th*, respectively.

A group averaged (n = 6) sagittal, axial, and incidence angle of each ROI are shown in Fig. 2. The group averaged angle of *Cp* (11.14 ± 2.52°) was 2.7 times higher than that of *Th* (4.13 ± 1.95°), with p = 0.667 × 10^–15^ (Fig. 2a) in the sagittal view, indicating that the rat skull was slightly pitched forward when fixed on the stereotactic frame used in this study. In addition, the group averaged sagittal angle of lateral ROI (13.64 ± 2.52°) was 1.96 times higher than that of the medial ROI (6.96 ± 1.66°) in *Cp,* with p = 2.167 × 10^–7^, while the group averaged angle of lateral ROI (14.11 ± 2.51°) was 1.95 times higher than that of the medial ROI (7.24 ± 2.25°) in *Th,* with p = 8.269 × 10^–7^ (Fig. 2b). The higher angle of lateral ROI in the axial view indicates the hemispheroidal anatomy of the rat skull. The averaged incidence angle at *Cp* (14.65 ± 3.38°) was 1.38 times higher than the averaged angle at *Th* (10.56 ± 3.79°), with p = 0.891 × 10^–5^ (Fig. 2c). Highest incidence angle was found at R1 (20.34 ± 0.96°), and the lowest was at R8 (9.74 ± 0.81°).

### Acoustic simulation

**Figure 3:**
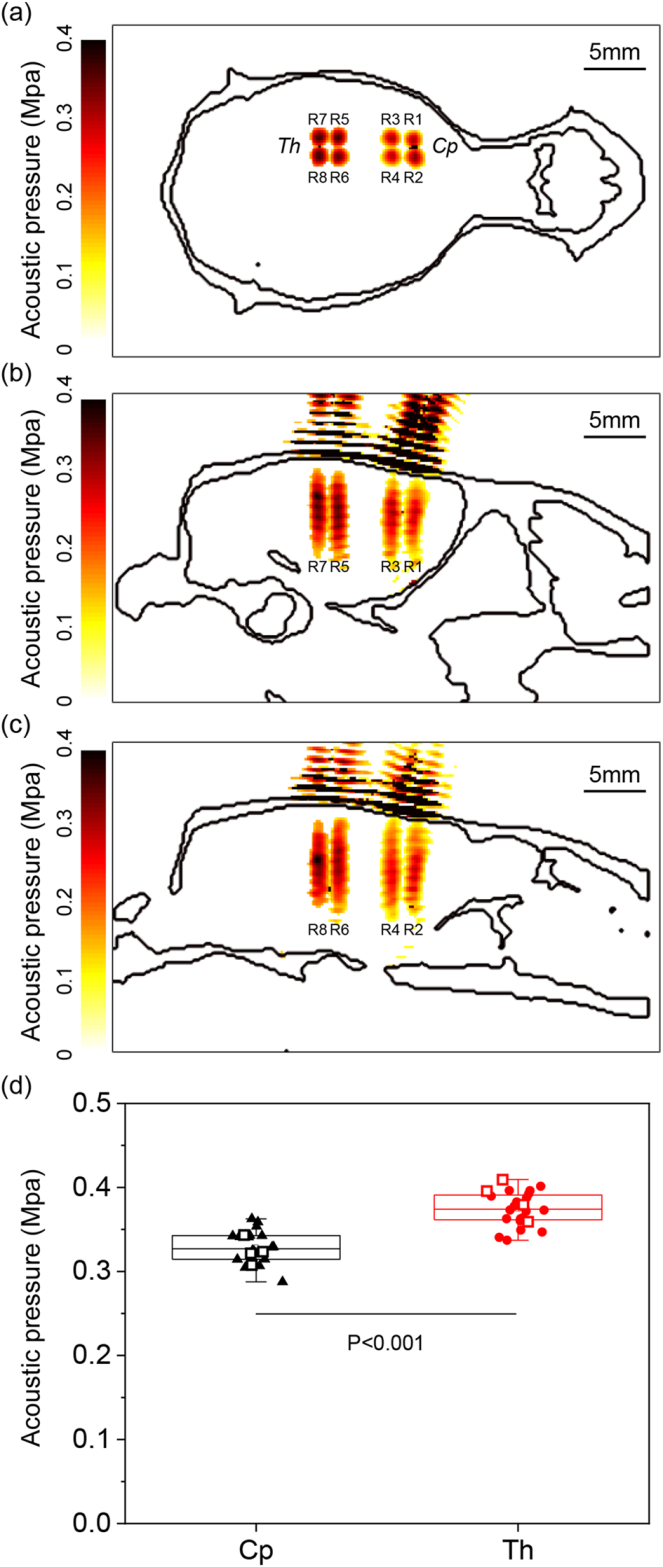
A representative acoustic pressure field simulation based on CT images on (**a**) coronal view, (**b**) lateral sagittal view, and (**c**) medial sagittal view. (**d**) Average acoustic pressure of *Cp* (triangle) and *Th* (circle). A representative simulation case is marked with a square. Images were generated using MATLAB R2017b (The Mathworks, USA).

**Figure 4:**
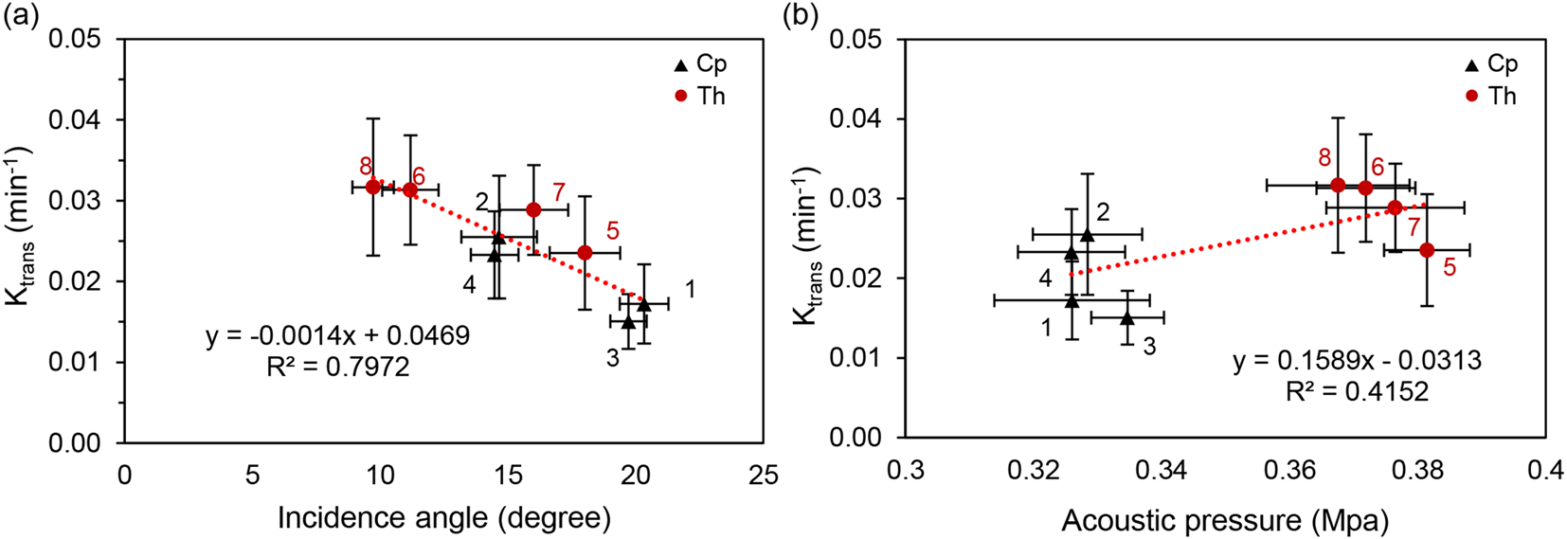
(**a**) BBB permeability K_trans_ and corresponding incidence angle of ROI. (**b**) BBB permeability K_trans_ and corresponding acoustic pressure of ROI. The dotted line represents linear fitting. The error bar in the x and y directions indicate standard deviation. Numbers indicate target regions, where 1 and 3 are in the lateral *Cp*, 2 and 4 are in the medial *Cp*, 5 and 7 are in the lateral *Th* and 6 and 8 are in the medial *Th*.

Figure 3 shows the representative acoustic simulation result and group averaged (n = 6) maximum acoustic pressure based on the rat skull anatomy reconstructed from the CT images. A maximum acoustic pressure field of each ROI was superimposed in the coronal (Fig. 3a) and two sagittal (Fig. 3b: medial and Fig. 3c: lateral) views. In the sagittal view, the *Th* region shows higher acoustic pressure, both in the lateral (Fig. 3b) and medial (Fig. 3c) cut planes. An average group (n = 6) simulation result, which compares the average maximum acoustic pressure of *Cp* (0.32 ± 0.01 MPa) and *Th* (0.37 ± 0.02 MPa), is shown in Fig. 4d. The average maximum acoustic pressure of *Th* was 1.16 times higher than that of *Cp*, with p = 0.138 × 10^–11^.

### Correlation between BBB permeability, incidence angle and acoustic pressure

The BBB permeability (K_trans_) of eight different ROIs calculated from the DCE-MRI (n = 11) group was compared with the incidence angle and the acoustic pressure measured and simulated from the CT (n = 6) group as depicted in Fig. 4a and b, respectively. The K_trans_ decreases as the incidence angle increases with R^2^ = 0.7972 and increases as the acoustic pressure increases (R^2^ = 0.4152). The red dot marked ROIs in *Th* regions. ROIs were distinctively grouped according to the brain region and the relative location (medial and lateral). The largest incidence angle group showed the lowest K_trans_ (R1 and R3; *Cp*_out), while the smallest incidence angle group showed the highest K_trans_ (R6 an R8; *Th*_in). R2 and R4; *Cp*_in, R5, and R7; *Th*_out were also grouped and positioned in the mid-range of the incidence angle and K_trans_. A correlation between K_trans_ and sagittal or axial angles showed a similar negative relationship with an R^2^ of 0.6941 and 0.3498, respectively. In addition, the average group mean K_trans_ (means of 5 slices in which K_trans_ was measured) linearly decreases with an increase in the incidence angle (R^2^ = 0.7664), similar to the maximum K_trans_. (Supplementary information S.1).

### Tissue staining

**Figure 5:**
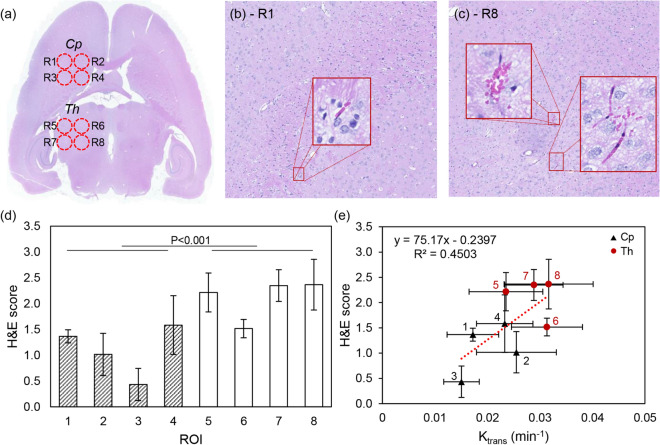
(**a**) A representative H&E staining image of eight different ROIs and 20 × magnified images of (**b**) R1 and (**c**) R8, respectively. A subset image shows the clusters of RBC. (**d**) The averaged H&E score of each ROI. (**e**) A correlation between the averaged H&E score and the corresponding K_trans_. Dashed line indicates the linear fitting curve with R^2^ = 0.4503. *Cp* and *Th* are marked with a triangle and circle, respectively.

Two different observers scored H&E histopathological images from a randomly selected case in the DCE-MRI group (n = 11), as described in the Methods—Histology section, by counting the red blood cell (RBC) at eight different ROIs (Fig. 5a). A representative H&E staining result of *Cp* (R1) and *Th* (R8) shows no significant region cavities (Fig. 5b,c, respectively). A few RBCs were observed, but no RBC clusters (n > 5) were found in *Cp* (R1 ~ R4). A few RBC clusters were observed in *Th* (R5 ~ R8). The average H&E score of *Cp* was 1.1 ± 0.89 and was 20% smaller than the average H&E score of *Th* (2.11 ± 0.79), with p = 0.000184 as shown in Fig. 5d. Here, R8 showed the highest H&E score of 2.37 ± 0.49, and R3 showed the lowest score of 0.43 ± 0.31. In addition, the H&E images were scored higher when K_trans_ was high, with R^2^ values of 0.4503 as shown in Fig. 5e.

## Discussion

This study aimed to investigate the degree of local permeability differences after FUS-induced BBBD at two representative ROIs in the rat brain (*Caudate putamen* and *Thalamus*). These two regions are widely used in pre-clinical rodent BBB experiments, as the *Cp* region (or striatum) involves motor function, and therefore has a possible application for Huntington’s and Parkinson’s disease treatment^7,8^. Meanwhile, the *Th* region located dorsal to the hippocampus is a potential target for Alzheimer’s disease treatment^13,20^. Recent advances in acoustic monitoring and feedback-controlled sonication demonstrated in a pre-clinical and clinical study may mitigate location-specific variability and safely control the BBB permeability^21–25^. Yet even with the feedback-controlled sonication, inter- and intra-subject variation was reported, hence understanding the sources of variability is important^21,26^.

In this study, a fixed acoustic power of corresponds to 0.65 MPa was used to sonicate the *Cp* and *Th* regions, yet the averaged K_trans_ of *Cp* was 33.1% smaller than the averaged K_trans_ of *Th,* as shown in Fig. 1f. In the previous study^14^, *Cp* and *Th* regions also had different MRI signal enhancement and leakage of trypan blue when sonicated under the same acoustic pressure, which indicates a difference in the level of BBB disruption. Considering these local differences, several previous pre-clinical studies^13,27^ used a higher level of acoustic power at *Cp* to achieve a similar level of BBB disruption, which resulted in a similar (around twofold) increase of chemotherapeutic substance DOX delivery compared to the contralateral hemisphere of both *Cp* and *Th*. The local concentration of DOX delivered to the sonication target was linearly correlated with the MRI signal enhancement and the BBB permeability K_trans_^14,28^. In addition, several attempts were made to enhance the local delivery of the chemotherapeutic agents by using multiple sonications^29^ or additional sonication without microbubbles before the main sonication^27^ and resulted in 26.08% (double sonication) and 75% (triple sonication) of DOX delivery increment. When compared to these attempts, a 33.1% difference in K_trans_ induced by the target’s characteristic reported in this study cannot be neglected as the following outcomes of the chemotherapeutic treatment may vary due to the DOX delivery difference^30^.

As shown in the H&E staining results (Fig. 5), no tissue damage except a few extravasated RBCs near blood vessels was found in the *Cp* region. When scored by two different observers, the average score measured in a representative sample between *Cp* and *Th* was statistically significant (p < 0.001) and linearly correlated with the BBB permeability measured in the sonication group at given ROIs (n = 11). Although enhanced BBB permeability can improve the delivery of the target substances, excessive acoustic energy can induce damages, such as extravasation, intracerebral haemorrhages, tissue damages, and edema^11,31^. Additionally, a recent study by Kovacs et al.^32^ demonstrated possible inflammation after the pulsed FUS treatment. As shown in Figs. 1 and 2, the regional differences in BBB permeability, as well as the incidence angle of *Cp* and *Th,* were statistically significant regardless of the variation between the individual ROIs and subjects. Differences in the degree of BBBD can be explained by the transmission of ultrasound energy, as shown in this study acoustic simulation’ results, where the averaged acoustic pressure of the focal area was lower at the *Cp* region compared to the *Th* region (Fig. 3). In our previous study, a free field ultrasound pressure distribution was measured using the Acoustic Intensity Measurement System (AIMS III, ONDA, USA) with and without a rat skullcap, in which the rodent skull insertion loss was 53.84 ± 0.91%. Using the identical transducer and experimental system, the group average acoustic pressure simulated in the current study was 50.58 ± 2.83 and 57.59 ± 0.13% of insertion loss at *Cp* and *Th*, respectively which are comparable with the previous study^19^. Moreover, the K_trnas_ and incidence angle of individual ROIs showed a strong correlation (R^2^ = 0.7972) (Fig. 4a). However, it is important to note that these measurements were not made on the same animal, hence inter-subject variation may exist. In addition, the skull’s inhomogeneity was not reflected in the current acoustic simulation, which is known to affect the precision of the ultrasound wave propagation especially in the human model^7,8^. However, the ultrasound wavelength (2.52 mm in the skull) of 1.113 MHz used in this study is larger than the typical rat skull’s thickness (less than 1 mm). The effect of homogeneous assumption may not be significant here than in the human skull (6.5 ~ 7.1 mm).

According to a previous study by Park et al.^33^, the averaged reflection coefficient inherited by the angle of incidence showed an inverse-linear relationship with acoustic pressure. In addition, another previous study by White et al.^34^ reported a decrease of the acoustic power transmitted via the skull of up to 30° of incidence angle, depending on the frequency. They also reported that there is a shear mode conversion during trans-human skull ultrasound propagation, especially in the high incidence angle. Thickness and curvature of the rat skull differ from the human skull, and the incidence angle in this study ranged from 9.73 ± 0.81 ~ 20.33 ± 0.96°. Current simulation results did not consider the shear mode conversion to simplify the simulation, and therefore warrants further studies. It is evident that the increased reflection owing to the incidence angle reduces the acoustic pressure and results in a decreased K_trans_. Some studies reported that an increase in the incidence angle might also affect the formation of the standing wave, especially in the small-animal pre-clinical models owing to their small cavity size. Acoustic simulation case was additionally repeated with a skullcap (bottom half removed) to analyse the standing wave formation and its effect on the acoustic pressure field. The formation of standing waves was noticeable in the acoustic pressure map compared to simulation case with the whole skull cavity. The maximum acoustic pressure in *Cp* and *Th* were increased 6.31 ± 3.87% and 6.95 ± 1.78%, respectively compare to the bottom half removed case. This suggests that the standing wave formation may also effect the local differences in BBB, especially for the small cavity subjects. (Supplementary information S.2).

Regardless of the high correlation between the permeability and incidence angles, this study has a few limitations that should be considered in future work. First, the comparison was not done on the same animal and the same transducer-skull registration, but from the same regions measured in different animal groups. However, the ear and tooth of the rat were tightly fixed to the stereotactic frame, and MR and CT images were sliced parallel to the animal bed, which fixes the transducer-skull orientation. In addition, regional differences in the permeability and incidence angles were statistically significant within the study group, which explains the correlation between these two parameters. Second, there could be another physiochemical effect that has not been considered in this study. Skull thickness is also known to affect the overall acoustic transmission in small animal models. Similar to this study, Gerstenmayer, et al.^6^ reported that the front part (*Cp* region of this study) showed a higher transmission rate compared to the middle part (*Th* region). Meanwhile, skull thickness in those two regions was identical within the subject. The size and density of the microvessel may also play an important role, as the FUS treatment involves the cavitation forces from the microbubble within the microvessel of the targeted area. According to a previous study, the interdependency of local capillary density in rat brains showed 460 ± 12.1 capillary sections per mm^2^ in *Th* and 363 ± 10.1/mm^2^ in *Cp*^35^. However, this aspect was not addressed in this study. In addition, chemotherapeutic agent delivery was not performed in this study, as it is previously known to be linearly related to BBB permeability. Although a wide range of incidence angles should be tested, for an easier and robust experiment the incidence angles of only two different ROIs were used in this study. Other parameters, such as tissue attenuation and local microbubble distribution attributed from the local differences in the size and density of micro-vessel were not controlled in this study, and their effect should be validated in future studies.

In conclusion, this study demonstrates a negative linear relationship between the incidence angle and the degree of permeability in vivo, mainly owing to the decrease in the acoustic power in a healthy rodent model. As the degree of permeability increases, a few extravasated RBCs near blood vessels were observed, but without any significant tissue damage. Even though the incidence angle is a primary source of the local differences in pre-clinical rodent experiments, tissue properties, including attenuation coefficient, capillary size, and density, may affect the degree of BBBD. Further studies on these will be beneficial for accurate and safe control of BBBD.

## Methods

### Animals

All experiments were conducted following procedures approved by the Daegu-Gyeongbuk Medical Innovation Foundation (DGMIF) Institutional Animal Care and Use Committee (IACUC, DGMIF-19100701-00). All procedures and animal handling were carried out following the ethical guidelines for animal research, with no pain or suffering of the animal. Animals (18 male Sprague–Dawley rats; eight weeks old; weighing 330 ± 28 g, Orient Bio Inc., Seongnam, Korea) were anesthetized with a mixture of Zoletil (35 mg/kg) and Rompun (5 mg/kg) before all experiments. The rats used in this study were randomly divided into two different experimental groups for measuring BBB permeability (n = 11) and incidence angle (n = 6).

### Focused ultrasound sonication

**Figure 6:**
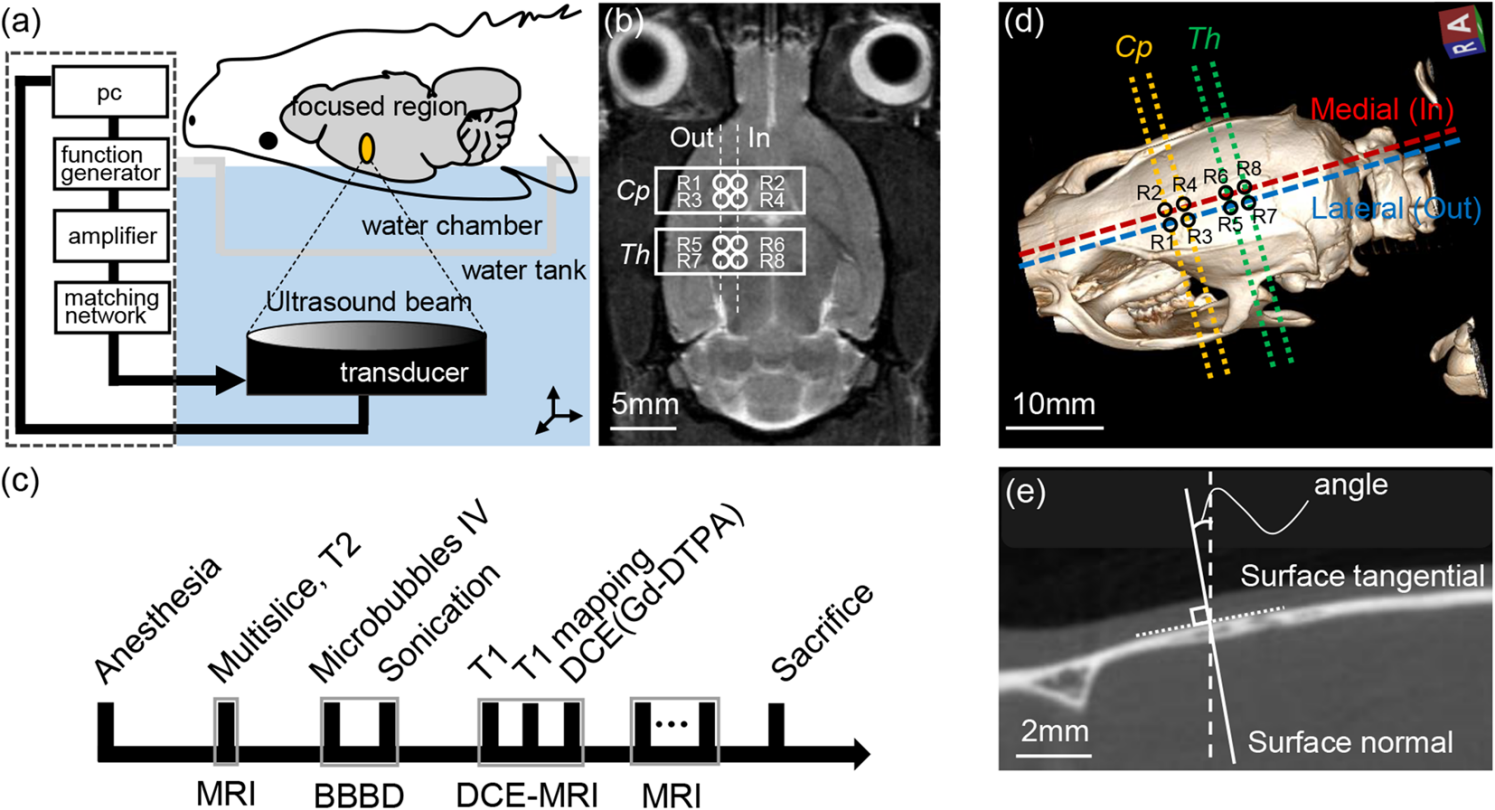
(**a**) A schematic of focused ultrasound blood brain barrier disruption system. (**b**) A T_1_-weighted MR image that represents 8 different sonication regions (*Cp*: R1 ~ R4, *Th*: R1 ~ R4). (**c**) An experimental protocol, which includes anaesthesia, sonication target guide by MRI (T_2_-weighted image), sonication, dynamic contrast-enhanced MRI, and sacrifice. (**d**) 3D volume rendering of a rat skull and the sagittal (dashed) and axial (dotted) plane for the angle measurement. Yellow and green dotted lines indicate *Cp* and *Th*, while red and blue dashed lines indicate medial and lateral ROIs, respectively. (**e**) Angle between the surface normal (solid line) derived from the surface tangential (dotted line) and the sonication path (dashed line) were measured as the sagittal and axial angles. 3D volume rendering image was generated using RadiAnt DICOM viewer 4.6.5 64-bit (Medixant Company, Poland).

A 10 ms burst sonication at a 0.65 MPa peak rarefaction focal pressure (PRFP) measured in a free water condition with 1 Hz pulse repetition frequency (PRF) for 120 s was delivered to the targeted area (*Cp* and *Th*) of rat brains in a supine position as shown in Fig. 6a. The ultrasound system and the experimental setup used in this experiment were identical to the previous study, where the free field ultrasound pressure distribution was measured using the Acoustic Intensity Measurement System (AIMS III, ONDA, USA)^36^. In brief, rat brains for BBB disruption were sonicated using a pre-clinical MRI guided Focused Ultrasound (MRgFUS) system (RK-100, FUS Instruments, Toronto, Canada). A spherically curved single-element piezoelectric transducer (FUS Instrument, Toronto, Canada) with a diameter of 75 mm, focal length of 60 mm, and a resonant frequency of 1.113 MHz was used for the sonication. Every four locations in the *Cp* (R1 ~ R4) and *Th* (R5 ~ R8) regions in the right hemisphere were set as the sonication targets, where the centres of targets were 1.5 mm apart from each other at each region of the brain. The reference region for *Cp* (R2) was 1.5 mm right and 1.5 mm anterior to the *bregma*, while the reference region for *Th* (R6) was 1.5 mm right and 3.5 mm anterior to the *lambda* (Fig. 6b). Focal spots were located 5 mm under the brain skull. Each sonication was applied synchronously to an intravenous injection of microbubble (Definity, Lantheus Medical Imaging, N.Billerica, MA), which consists of a C_3_D_8_ gas encapsulated by an outer phospholipid shell. The solution was diluted 50-fold in saline and intravenously injected at a dose of 10 μL/kg. Microbubbles with less than two hours of activation were used (manufacturer’s guidelines < 12 h) to assure a similar level of microbubble concentration for each experiment. The order of the sonication group was randomized (4 targets sonicated in *Cp* followed by 4 targets in *Th* and vice versa) in order to minimize the experimental bias. A five-minute delay was allowed between sonication to clear the bubble from the system and minimize the increase of microbubble concentration during the second exposure based on previous studies^37,38^.

### Magnetic resonance imaging

The imaging was performed using the 9.4 T pre-clinical MRI system (BioSpec 94/20 USR, Bruker, Ettlingen, Germany). An 86 mm inner diameter volume coil was used for RF transmission and signal reception. A 2D rapid-acquisition with relaxation enhancement (RARE) T_1_-weighted pulse sequence (echo time; TE = 6.5 ms, repetition time; TR = 1500 ms, field of view; FOV = 40 × 40 mm^2^, matrix size = 256 × 256, bandwidth = 100,000 Hz, RARE factor = 4 and slice thickness = 1.5 mm) in coronal view was used to detect the local BBB disruption. A 2D RARE T_2_-weighted pulse sequence (echo time; TE = 33 ms, repetition time; TR = 2500 ms field of view; FOV = 40 × 40 mm^2^, matrix size = 256 × 256, bandwidth = 36,765 Hz, RARE factor = 8 and slice thickness = 1.5 mm) in coronal view was used to guide the sonication procedure, similar to previous studies^13,27,28,36^.

A RARE with variable repetition time TR (RARE VTR) pulse sequence (TE = 7.5 ms, TR = 160, 180, 200, 250, 300, 400, 500, 600, 700, 800, 900, 1000, 1200, 1500, 1800, 2000, 2500, 3000, 3500, 4000, 6000, and 12,000 ms, FOV = 40 × 40 mm^2^, matrix size = 128 × 128, bandwidth = 78,125 Hz, RARE factor = 1, and slice thickness = 1.5 mm) in coronal view was used for the T_1_ mapping before sonication. Ten pre-contrast sets of fast low-angle shot (FLASH) DCE-MRI (TE = 1.3 ms, TR = 24.4 ms, FOV = 40 × 40 mm^2^, matrix size = 128 × 128, bandwidth = 85,227 Hz, slice thickness = 1.5 mm) followed by an additional 90 sets under the intravenous administration of CA were acquired in a coronal view with a temporal resolution of 6 s for ten minutes to calculate the BBB permeability. A total of five slices with a 1.5 mm gap were used for T_1_ mapping and DCE-MRI, while the middle slice (3rd) was positioned at the focal area of the sonication (~ 5 mm under the skull). The permeability (K_trans_) of eight individual sonicated areas at five different slices, which mostly covered the brain tissue (4-pixel circle diameter corresponding to the 1.5 mm focal area of the transducer (3.2 pixels/mm), was calculated using the Patlak model^39^ (Eq. 1).

1 $${C}_{t}\left(t\right)={K}_{trans}{\int }_{0}^{t}{C}_{p}\left(\tau \right)d\tau +{V}_{p}\cdot {C}_{p}\left(t\right)$$

Here, t, τ, V_p_ C_t_(t) and C_p_(t) indicate the time step, variable of integration, plasma volume, and temporal variation of the contrast agent concentration in the tissue and the plasma, respectively. The C_p_(t), also known as the arterial input function (AIF), was individually measured in a feeding vessel close to the ROI^40^, and the capillary haematocrit level was adjusted to 45%^41^. The maximum K_trans_ in this study represents the maximum value of the five slices of the volume of interest (1.5 × 1.5 × 7.5 mm^3^), while the mean K_trans_ indicates the average of K_trans_ measured in the five different MRI slices. An overall experimental protocol includes anaesthesia, sonication target guide by MRI (T_2_ image), sonication, dynamic contrast-enhanced MRI, and sacrifice as shown in Fig. 6c.

### Computational rat model and acoustic simulation

For the acoustic simulation, the computational rat model used in this study was obtained using a pre-clinical micro-CT (R_mCT2, Rigaku, Japan) images of rats (n = 6) with a spatial resolution of 0.082 mm/pixel. A volume of interest was set parallel to the transducer in order to replicate the orientation of the transducer with respect to the rodent skull reconstructed in 3D. The relative orientation was confirmed using a clinical CT (Siemens Biograph mCT, Germany) image of the entire sonication system including the water chamber, transducer, animal bed, and animal fixed on a stereotaxic frame. The computation model was constructed as three parts (skin, skull, and brain) and was extracted using CT Hounsfield units of the micro-CT images. Physical properties of the medium used in the simulation are given in Table 1.

**Table 1:** Acoustic properties of computational rat model.

Material	Speed of sound (m/s)	Density (kg/m^3^)	Attenuation coefficient (Np/m/MHz)
Skin^42^	1624.0 623	1109	43.966
Skull^42^	2813.7	1908	113.36
Brain^42^	1546.3	1046	17.60
Water^43^	1482	1000	0.025

Acoustic simulations with the same transducer positions and targets used in the BBB opening experiment (guided by the *bregma* and *lambda* of 3D reconstructed rat skull) were performed to investigate the acoustic pressures and the focusing effects at the target in the brain. A commercial acoustic simulation software (Sim4Life, ZMT, Switzerland) was used to numerically solve the linear acoustic pressure wave equation (Eq. 2)

2 $$\rho \nabla \frac{1}{\rho }\nabla p - \frac{1}{{c^{2} }}\frac{{\partial^{2} p}}{{\partial t^{2} }} - \frac{\alpha }{{c^{2} }}\frac{\partial p}{{\partial t}} = 0,$$

where *ρ, p, c,* and *α* are the local density, acoustic pressure, speed of sound, and absorption, respectively, and *t* is the time. This simulation software was numerically and experimentally validated in free water as well as the transcranial conditions (sheep skull) using the ‘Gamma-method’ to judge the quality of simulation and measurement agreement^43–45^. The domain of the acoustic simulation performed in this study was 603 × 603 × 716 grid points, with a spatial discretization of ten points per wavelength (i.e., 0.133 mm). The pulse duration of sonication was set at 89 µs to reduce the computation time, and a time step of 27.3 ns was used to compute the simulation. The two sagittal (dashed) and four axial (dotted) views that pass each sonication region were computed using a multi-planar reconstruction (MPR) of the 3D skull feature (Fig. 6d). The angle between the surface normal and the transducer beam path was measured as sagittal and axial angles (Fig. 6e). The intra- and inter-observer variation of the incidence angle measurement was small (R^2^ = 0.8841 and 0.8197, respectively). The incidence angle calculated from the observer’s measurements showed good agreement (R^2^ = 0.7895) with the incidence angle directly measured from the 3D skull feature by averaging the normal vectors within the ultrasound focal area (1 mm^2^).

### Histology

A brain of a rat was extracted and embedded in paraffin for the 4 μm sectioning in the axial view (perpendicular to the direction of ultrasound beam propagation) to evaluate the histological effects of the sonicated region. Three representative planes, including the focal area of the ultrasound, were stained with haematoxylin and eosin. Histological scoring was performed on eight different sonicated regions as well as its contralateral area of the opposite hemisphere. Each section was scored between 1 (0 < n < 5), 2 (5 < n < 10), and 3 (n > 10) according to the number of red blood cells by two different observers. The inter-observer variation was minimal, with the bias of − 0.126 and the 95% limit of agreement of 0.391 and − 0.647 (R^2^ = 0.9394).

### Statistical analysis

The statistical analysis was performed using SPSS software (IBM SPSS Statistics; IBM Corp., Armonk, NY, USA) by two researchers blind to the animal assignment. All data was presented as the mean ± standard deviation and analysed with the equal variance two-tailed t-test.

## Supplementary information

Supplementary information.
