# Supplementary material for: A local difference in blood–brain barrier permeability in the caudate putamen and thalamus of a rat brain induced by focused ultrasound
Source: Sci Rep. 2020 Nov 6;10:19286. doi: 10.1038/s41598-020-76259-z (PMC7648079; doi:10.1038/s41598-020-76259-z)
Supplement: Supplementary file 1 — Supplementary information. [file 41598_2020_76259_MOESM1_ESM.pdf]

Supplementary information

A local difference in blood-brain barrier permeability in the *caudate putamen* and *thalamus* of a rat brain induced by focused ultrasound

Hyungkyu Huh[1], Tae Young Park[2], Hyeon Seo[1], Mun Han[1], Byeongjin Jung[1], Hyo Jin Choi[1], Eun-Hee Lee[1], Ki Joo Pahk [2], Hyungmin Kim[2*] and Juyoung Park[1*]

[1] Medical Device Development Center, Daegu-Gyeongbuk Medical Innovation Foundation, Daegu, Korea

[2] Center for Bionics, Korea Institute of Science and Technology, Seoul, Korea

[3] Division of Bio-Medical Science & Technology, KIST School, Korea University of Science and Technology, Seoul, Korea

*Corresponding authors

Hyungmin Kim, Korea Institute of Science and Technology, Seoul, Korea, Tel: +82 02-958-5695; E-mail address: hk@kist.re.kr

Juyoung Park, Medical Device Development Center, Daegu-Gyeongbuk Medical Innovation Foundation, Daegu, Republic of Korea, Tel: +82 053-790-5590; E-mail address: jyp@dgmif.re.kr

Keyword: Focused ultrasound, Blood brain barrier disruption, Incidence angle, Dynamic contrast enhanced MRI, Acoustic simulation

S.1 Axial and sagittal angle comparison with the brain permeability

A sagittal and axial angle according to the group averaged brain permeability (K_{trans}) was compared. Both angles showed a negative relationship with the brain permeability, and the corresponding R^2 was 0.6941 and 0.3498 for the sagittal and axial angle, respectively (Fig. S1). A group averaged (n=11) mean K_{trans} shows a negative linear relationship with the incidence angle with R^2=0.7664 (Fig. S2).

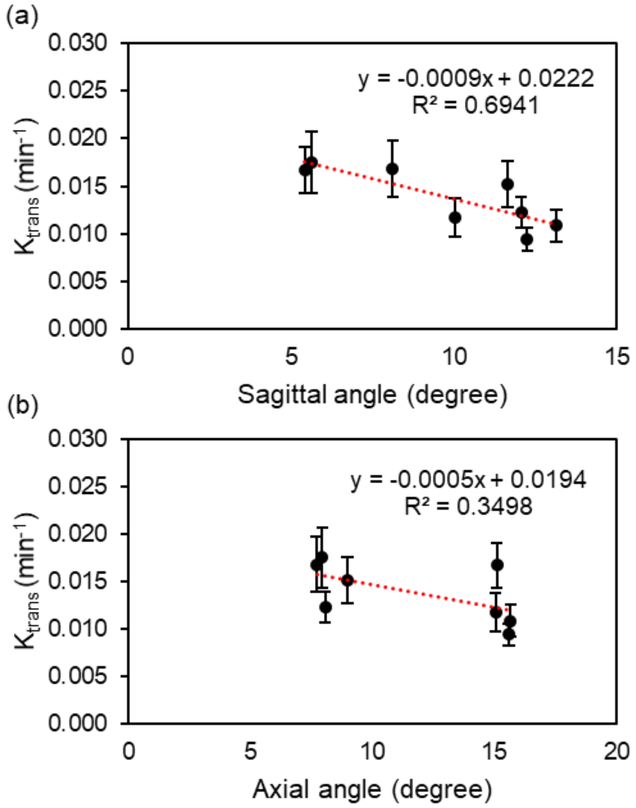

Fig. S1. The group averaged (n=11) maximum K_{trans} according to the angle measured at a) sagittal view and b) axial view. The dotted line indicates linear fitting between the permeability and angles with R2 of 0.6941 and 0.3498 for the sagittal and axial angle, respectively.

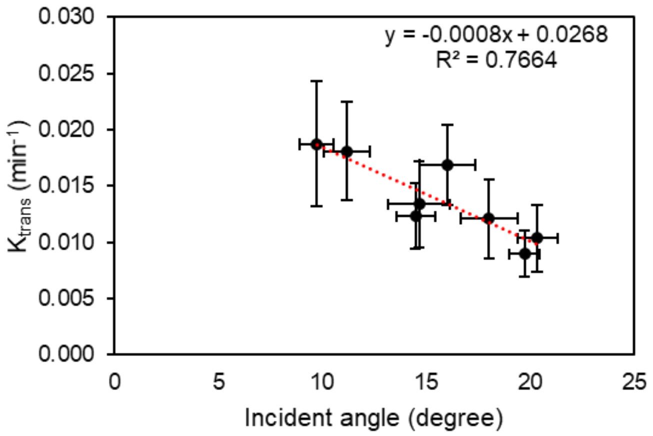

Fig. S2. The group averaged (n=11) mean brain permeability (K_{trans}) according to the incident angle. The dotted line indicated linear fitting with R^2 of 0.7664.

S.2 An acoustic pressure map of whole skull and bottom half removed skull

A two acoustic simulation was conducted on a representative case keeping the whole skull cavity and removing the bottom half in order to observe the formation of a standing wave. As shown in the Fig.S3b and c, wavy patterns that indicate the formation of the standing wave is observed. On the other hand, the smooth acoustic pattern was shown when the bottom half of the skull is removed prior to the simulation. However, the location of the maximum acoustic pressure (a cross mark) was not affected by the standing wave formation, and the differences in the averaged pressure were less than 7% (6.31±3.87% for *Cp* and 6.95±1.78% for *Th*).

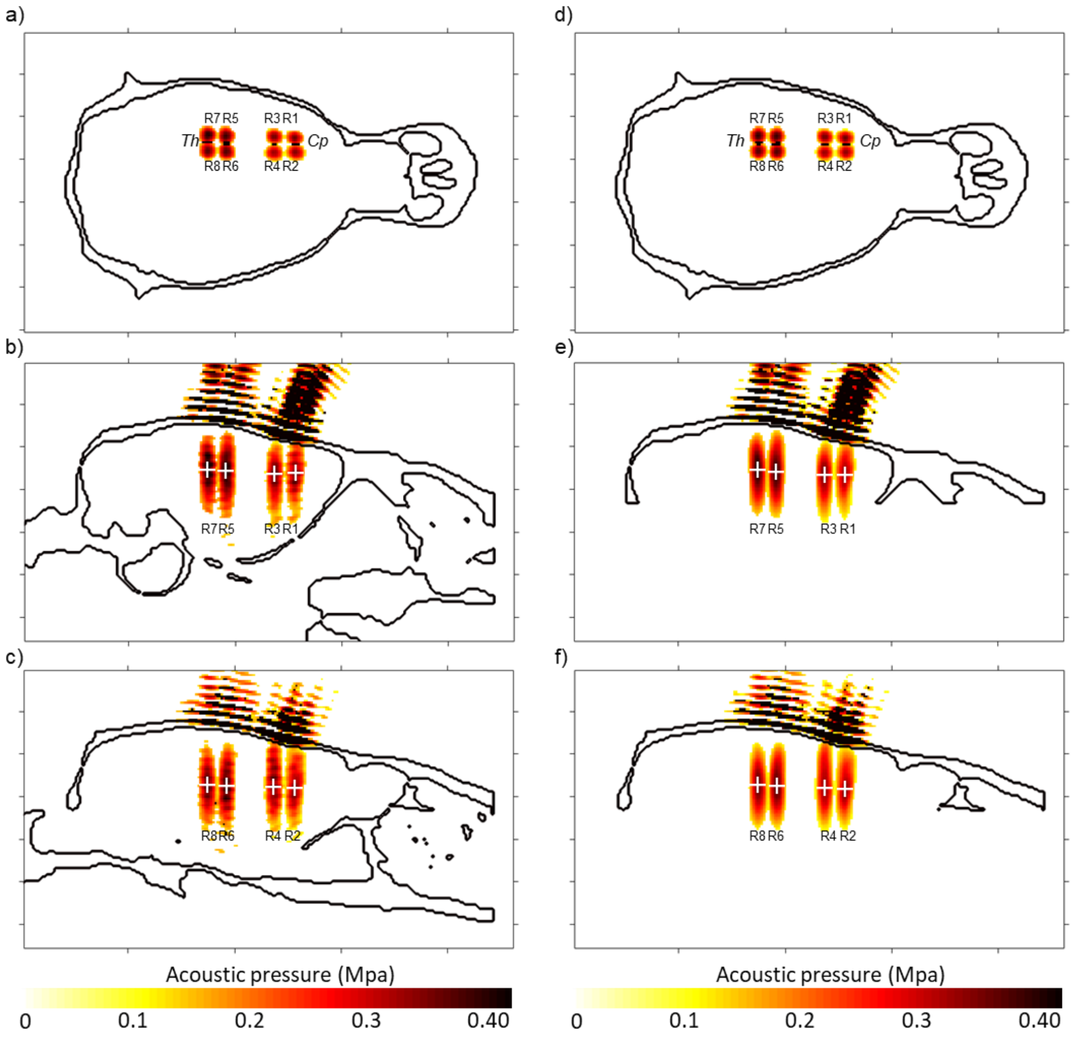

Fig S3. A representative superimposed acoustic pressure map with the bottom half shown in a) a coronal, b) lateral-sagittal and c) medial-sagittal view. A acoutic pressure map without the bottom half in d) a coronal, e) lateral-sagittal and f) medial-sagital view. A cross indicates the location of the maximum acoustic pressure.
